# Giant fibrous polyp issuing from the uterine cervix: A case report

**DOI:** 10.1016/j.radcr.2022.02.070

**Published:** 2022-03-26

**Authors:** Daoud Bentaleb, Leila Noureddine, Vianney Ndayishimiye, Mariam Mourabbih, Abderrahmane Mellouki, Mouna Sabiri, Ghizlane Lembarki, Fatiha Essodegui, Nisrine Bennani Guebessi

**Affiliations:** a Central Service of Radiology, Ibn Rochd University Hospital, Faculty of Medicine and Pharmacy of Casablanca, Casablanca, Morocco; b Gynaecology and Obstetrics Service, Ibn Rochd University Hospital, Faculty of Medicine and Pharmacy of Casablanca, Casablanca, Morocco; c Anatomopathology Service, Ibn Rochd University Hospital, Faculty of Medicine and Pharmacy of Casablanca, Casablanca, Morocco

**Keywords:** Fibrous polyps, Giant, Pedunculated, Magnetic resonance, Ultrasonography, Cervix

## Abstract

Fibrous uterine polyps are very common in women during or after menopause, and less often seen in women of child-bearing age, with a maximum of frequency between 40, and 49 years. They can have various locations in the uterus depending on the patient's age, mostly the body, and fundus. We report a rare case of cervical localization of a pedicled fibrous polyp issuing from the cervix, in a 44-year-old female patient with 3 living children and a history of miscarriage, who had been presenting breakthrough bleeding, and pelvic pain for 3 months. It is essential to remind young radiologists of the different presentations of fibrous polyps, how to explore them better and when to fear malignancy and thus insist in a histologic study, in order to help clinicians to choose the most adequate treatment option.

## Introduction

Fibrous uterine polyps are very common in women during or after menopause, and less often seen in women of child-bearing age, with a maximum of frequency between 40, and 49 years [1]. They can have various locations in the uterus depending on the patients’ age, mostly the body, and fundus [1]. We report a rare case of cervical localization of a pedicled fibrous polyp issuing from the cervix, with a striking radiological, and anatomopathological iconography.

## Case presentation

We report the case of a 44-year-old female patient with 3 living children and a history of miscarriage, who had been presenting breakthrough bleeding, and pelvic pain for 3 months.

Speculum examination showed a prolapsed mass that bleeded at contact. Cervix was not visible.

**Fig. 1:**
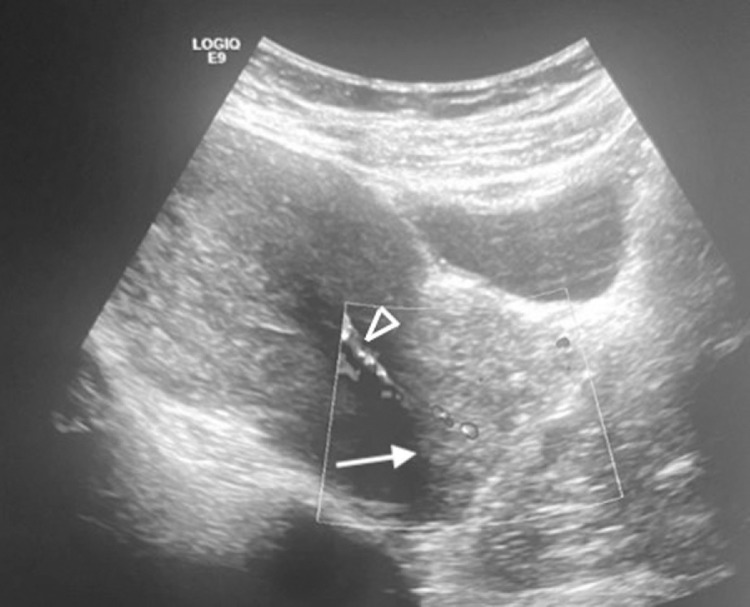
Median sagittal view of the suprapubic pelvic ultrasonography done on our patient, showing a cervical mass of heterogenous echopattern (white arrow), with visualization of the feeding artery in Doppler mode (arrowhead).

The patient was then addressed to our structure, first, for a suprapubic pelvic ultrasonography, which showed a voluminous cervical mass of heterogenous echopattern, and Doppler signal (Fig. 1).

**Fig. 2:**
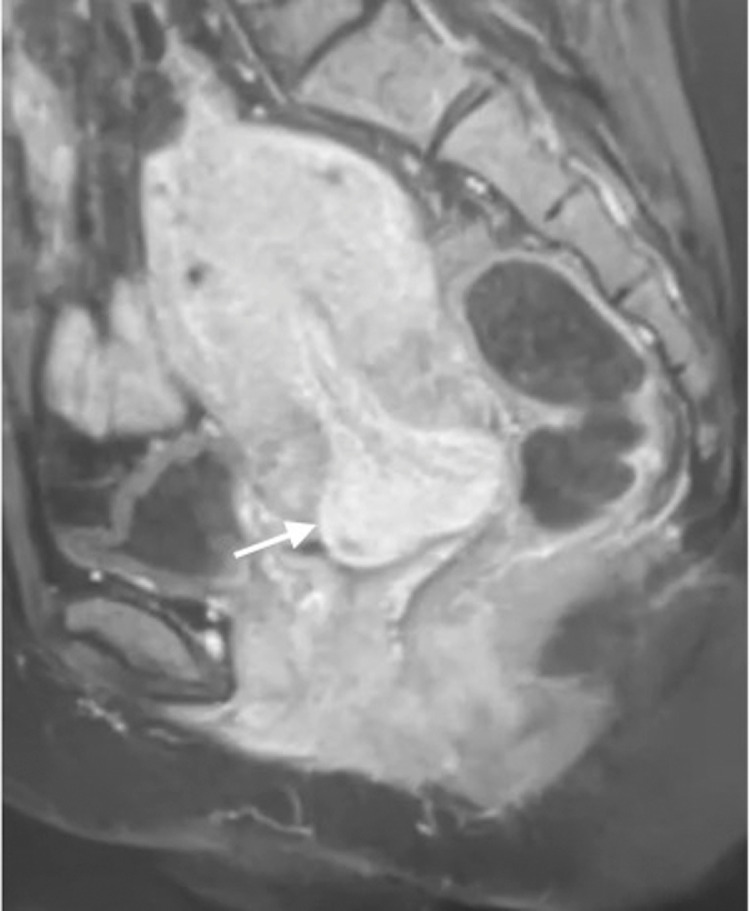
Sagittal plane T1-weighted fat suppressed postcontrast pelvic MR section, showing a voluminous cervical polyp issuing from the cervix, with intense, homogenous contrast enhancement (white arrow).

She was later on addressed for a complimentary pelvic MRI, which showed a voluminous endometrial polyp issuing from the uterine cervix (Fig. 2) in hyposignal in T1 weighting, hypersignal in T2 weighting, heterogenous signal in the Diffusion sequence, with marked contrast enhancement after gadolinium injection. We concluded that it was preferrable to confront this mass to its histologic findings because of its size and heterogenous Diffusion signal.

**Fig. 3:**
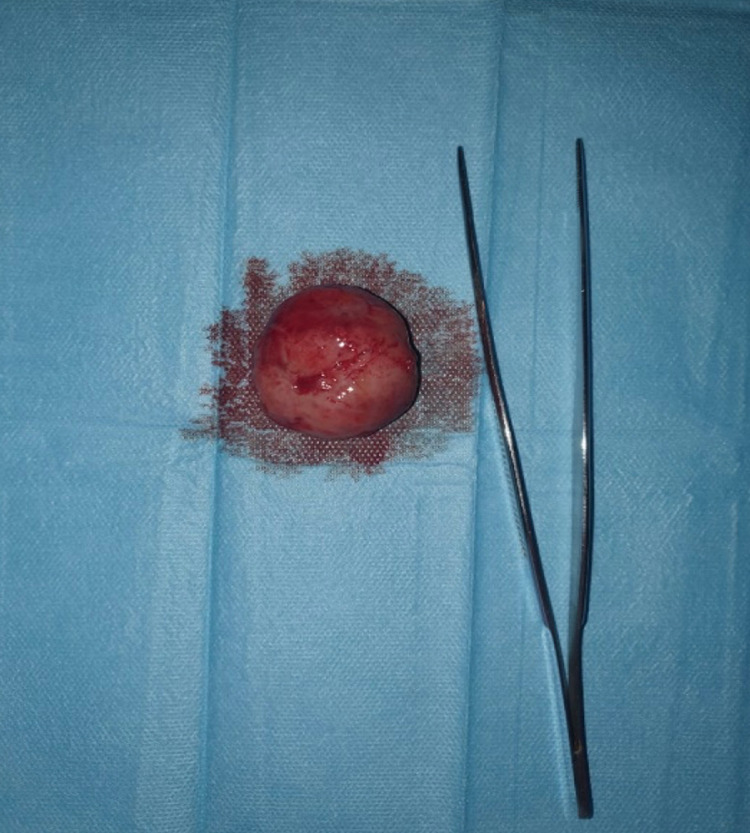
Photograph of the surgically excised tissues, the poly measures 4 cm of diameter.

The patient was consequently operated on in the Gynecology and Obstetrical structure of our hospital, with a total excision of the mass (Fig. 3), and simple postoperative aftermath.

**Fig. 4:**
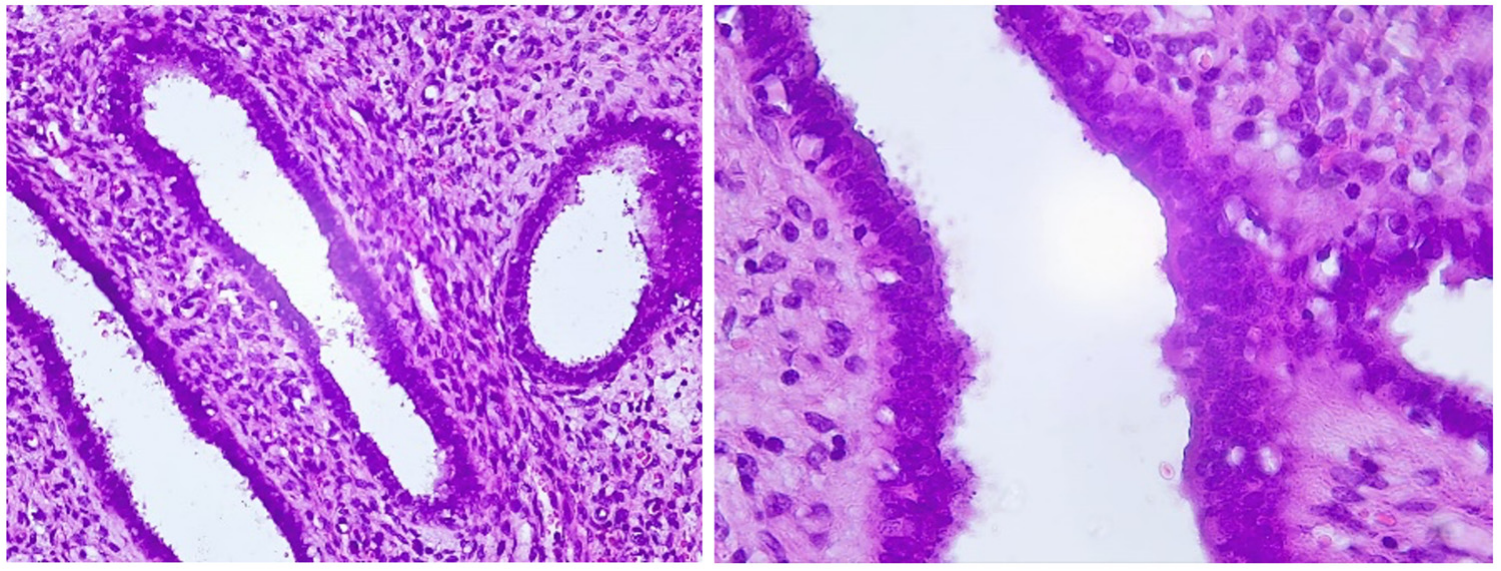
Low-power (left) and higher-power (right) magnification of the excised tissues, with Hematoxylin and eosin stain, showing adenomatous proliferation with marked crowding, and loss of stroma.

Histologic findings provided us with the final diagnosis of a fibrous polyp of the uterine cervix (Fig. 4).

## Discussion

Endometrial fibrous polyps are a frequent gynecologic condition that can cause abnormal uterine bleeding, pelvic pain or infertility, or remain silent, and get discovered by chance at the occasion of a pelvic imaging examination in an unrelated context. This condition is due to an abnormal, generally localized, endometrial intrauterine overgrowth that can be single or multiple, and may be sessile or pedunculated [2].

The reported prevalence of endometrial polyp varied widely between 7.8% and 34.9%, depending on the study, and the definition of a polyp. It appears to be more frequent as one advances in age, especially in post-menopausal women (11.8% vs 5.8% in premenopausal women); however, there might be a selection bias since postmenopausal vaginal bleeding is more likely to be investigated [2].

The most common symptoms associated with this condition are abnormal uterine bleeding, but also menorrhagia, irregular menses, postcoital bleeding (especially for polyps issuing from the cervix, like our reported case), intermenstrual bleeding, or infertility, although they can remain asymptomatic for years [2].

Microscopically, endometrial polyps are typically a mixture of dense fibrous tissue (stroma), large and thick-walled vascular channels, and glandular spaces of varying shapes and sized, covered by a surface epithelium [1,3].

Polyps issuing from the uterine cervix represent 23% of all their locations on the uterus [1].

As for diagnostic imaging procedures, many are contributive in exploring uterine fibromas of usual locations.

The first one is, without surprise, pelvic ultrasonography. It generally shows a hyperechoic lesion with regular contours, within the uterine lumen, outlining the endometrial walls on which it rests, surrounded by a thin hyperechoic halo. Doppler mode can be very beneficial, especially when it is actually able to show the feeding artery [2,4].

If the lesion's size is too small to be correctly assessed with suprapubic sonography, one can consider the using the transvaginal way. Some authors even say it can be equivalent to hysteroscopy in this indication [5].

Another imaging feature used to assess such lesions is sonohysterography, with the use of saline infusion in order to better outline the endometrial cavity, and thus, the contours, size, and location of endometrial polyps [2].

However, in the particular case of polyps issuing from the uterine cervix, such imaging modalities are hardly useful because of the difficult access to the lesion. That is why, in this particular case, pelvic MRI can be very helpful.

A standard MRI protocol for exploring the pelvis in women with such pathologies constitutes in sagittal, oblique axial and oblique coronal slices in T1 and T2 weighting as well as Diffusion and contrast sequences. It is important to note that Diffusion sequences play an important role in differential diagnosis between endometrial cancer, polyp, hyperplasia, and physiological thickening [6].

Polyps appear essentially as large lesions filling the endometrial cavity or, in our case, issuing from the cervix, with variable T1 and/or T2 signal and contrast enhancement, isointense with the myometrium in Diffusion sequences with high ADC values [7].

As for histologic diagnosis, the best option available is hysteroscopy with guided biopsy as it allows us to visualize and remove polyps concurrently. Advances in technology permits specialists to even remove them under direct vision, in some cases [7].

This brings us to briefly present the different management options, which go from observation to conservative surgical, and radical surgical procedures. The choice of treatment will be based on the importance of the symptoms and their effects on the patient's daily life but also other factors such as malignancy risk, fertility issues, and operator's skills. Clinical outcomes after treatment of endometrial polyps are generally good, with a significant lessening of symptoms such as intermenstrual bleeding [2].

## Conclusion

Adenomatous uterine polyps are a quite common condition in women, with cervical pedunculated ones being rarer, and of quite a peculiar presentation. Radiologists should know about their different presentations, how to explore them better and when to fear malignancy and thus insist in a histologic study, in order to help clinicians to choose the most adequate treatment option. Our case's lies in its striking iconography and its positive follow-up.

## Patient consent

Written and informed consent for publication of the case was obtained from the patient.
